# Design and Characterization of Biomimetic Hybrid Construct Based on Hyaluronic Acid and Alginate Bioink for Regeneration of Articular Cartilage

**DOI:** 10.3390/pharmaceutics16111422

**Published:** 2024-11-07

**Authors:** Cristina Galocha-León, Cristina Antich, Beatriz Clares-Naveros, Ana Voltes-Martínez, Juan Antonio Marchal, Patricia Gálvez-Martín

**Affiliations:** 1 Department of Pharmacy and Pharmaceutical Technology, Faculty of Pharmacy, University of Granada, E-18071 Granada, Spain; galochacristina@gmail.com (C.G.-L.); beatrizclares@ugr.es (B.C.-N.); 2 Biopathology and Regenerative Medicine Institute (IBIMER), Centre for Biomedical Research, University of Granada, E-18100 Granada, Spain; cantich@go.ugr.es (C.A.); ana.voltes@gmail.com (A.V.-M.); 3 Instituto de Investigación Biosanitaria ibs.GRANADA, University Hospital of Granada, University of Granada, E-18100 Granada, Spain; 4 Department of Human Anatomy and Embryology, Faculty of Medicine, University of Granada, E-18012 Granada, Spain; 5 Excellence Research Unit “Modeling Nature” (MNat), University of Granada, E-18071 Granada, Spain; 6 BioFab i3D—Biofabrication and 3D (Bio) Printing Laboratory, University of Granada, E-18100 Granada, Spain; 7 R&D Human and Animal Health, Bioibérica S.A.U., E-08029 Barcelona, Spain

**Keywords:** hyaluronic acid, mesenchymal stromal cell, bioprinting, construct, cartilage tissue engineering

## Abstract

**Background/Objectives:** Three-dimensional bioprinting technology has enabled great advances in the treatment of articular cartilage (AC) defects by the biofabrication of biomimetic constructs that restore and/or regenerate damaged tissue. In this sense, the selection of suitable cells and biomaterials to bioprint constructs that mimic the architecture, composition, and functionality of the natural extracellular matrix (ECM) of the native tissue is crucial. In the present study, a novel cartilage-like biomimetic hybrid construct (CBC) was developed by 3D bioprinting to facilitate and promote AC regeneration. **Methods:** The CBC was biofabricated by the co-bioprinting of a bioink based on hyaluronic acid (HA) and alginate (AL) loaded with human mesenchymal stromal cells (hMSCs), with polylactic acid supporting the biomaterial, in order to mimic the microenvironment and structural properties of native AC, respectively. The CBC was biologically in vitro characterized. In addition, its physiochemical characteristics were evaluated in order to determine if the presence of hMSCs modified its properties. **Results:** Results from biological analysis demonstrated that CBC supported the high viability and proliferation of hMSCs, facilitating chondrogenesis after 5 weeks in vitro. The evaluation of physicochemical properties in the CBCs confirmed that the CBC developed could be suitable for use in cartilage tissue engineering. **Conclusions:** The results demonstrated that the use of bioprinted CBCs based on hMSC-AL/HA-bioink for AC repair could enhance the regeneration and/or formation of hyaline cartilaginous tissue.

## 1. Introduction

Articular cartilage (AC) tissue injuries remain a major challenge in orthopedic surgery since its regenerative capacity is limited, given its poor cellularity and avascular nature [1,2]. A range of options such as medication, rest, physiotherapy, and surgery are currently used to treat cartilage damage and repair [3]. Focal lesions of AC are surgically treated using techniques such as the microfracture, the abrasion arthroplasty, the debridement, the implantation of allografts, autologous chondrocytes (ACIs), matrix-induced chondrocyte transplantation (MACI), or autologous matrix-induced chondrogenesis (AMIC) [4,5,6,7,8]. Each of these methods has specific strengths and limitations [3,9,10]. Although the current clinical approaches have achieved limited success, these treatments often involve a limited recovery of joint function associated with the formation of inadequate tissue. Repair procedures after cartilage injury can lead to the excessive secretion and deposition of natural extracellular matrix (ECM) proteins that can result in fibrocartilage formation with lower mechanical strength than native hyaline cartilage [4,5,7,11,12,13]. Therefore, the regeneration of hyaline cartilage remains the fundamental goal in the treatment of cartilage injuries. Recently, tissue engineering has emerged as an interdisciplinary field focused on developing new therapeutic approaches for cartilage regeneration. This involves creating biological substitutes that can restore, maintain, or enhance the function of damaged tissue. Generally, this is performed by culturing the cells in a three-dimensional (3D) structure, which mimics the composition and architecture of the ECM of the native AC being developed in order to be locally implanted in the injured AC, trigging and supporting the regeneration process [14,15]. Three-dimensional bioprinting enables the manufacture of individualized scaffolds with controllable micro-structures that provide cells a suitable microenvironment, biochemical stimulus, and mechanical support, which would eventually facilitate cell behavior including growth, proliferation, and differentiation [16,17].

Bioinks, bioprintable materials used in 3D bioprinting processes, can be divided into two categories: scaffold-based bioinks and scaffold-free bioinks. In the scaffold-based bioink, cells are loaded into hydrogels or similar exogenous materials, which are then bioprinted to create 3D structures [18,19]. In contrast, scaffold-free bioinks are composed of aggregates such as tissue spheroids [20,21,22], cell pellet [23], and tissue strands [24], where cells are bioprinted without the necessity of an external biomaterial [18,19]. Numerous researchers have reported the manufacturing of biomimetic constructs from hydrogels loaded with living cells [25,26,27,28,29,30,31,32,33,34]. The hydrogels provide an appropriate biomimetic microenvironment for cell proliferation, migration, differentiation, and adhesion [35]. In addition, hydrogels are biocompatible and biodegradable and have rich water content, which supply a matrix that can resemble natural components of the native ECM of AC [26,27,36,37,38,39,40,41,42]. However, despite these advances, the use of hydrogels has limitations such as their variability to obtain adequate mechanical properties. For this reason, the bioprinting of biomimetic hybrid constructs by the simultaneous deposition of hydrogels loaded with living cells and supporting biomaterials may offer a novel and more suitable strategy generating 3D structures that mimic the structural and compositional heterogeneity of native AC [43,44].

The bioprinting of cartilage constructs is generally extrusion based, which is the most common and affordable bioprinting method [17,45,46]. Alternatively, inkjet [47,48], laser-induced forward transfer (LIFT) [49], and 3D bioprinting provide a higher resolution, though they come at a considerably higher cost [50]. Each of these methods has specific strengths, weaknesses, and limitations [51].

Among biomaterials used as cell carriers, hyaluronic acid (HA) is one of the most promising natural polymers for cartilage tissue engineering (CTE). In osteoarthritic chondrocytes, HA enhances cellular metabolic activity, thereby promoting biosynthesis at the cellular level [52,53,54], playing a critical role during chondrogenesis and maintaining cartilage homeostasis [55,56]. In addition, it has been demonstrated that HA plays a role in the migration and proliferation of chondrocytes through its interaction with specific cell surface receptors such as CD44 [57,58]. Several studies have demonstrated that HA improves cell functionality in both chondrocytes and mesenchymal stromal cells (MSCs), promoting the chondrogenic phenotype and hyaline-specific matrix deposition [32,59,60,61,62,63,64], as well as suppressing genes associated with cartilage inflammation [65]. Moreover, HA is one of the main glycosaminoglycan (GAGs) present in the cartilage ECM and exhibits suitable properties such as biocompatibility, biodegradability, and non-toxicity [66,67,68]. Despite these advantages, HA presents a limited gelation capacity, which results in poor rheological properties for maintaining cell suspension [69,70]. In this context, HA has been mixed with other biomaterials, such as alginate (AL), to develop more viscous formulations suitable for use as bioinks [71,72]. AL is considered a gold standard in CTE due to its easy handling, excellent rheological properties for bioprinting, fast gelation kinetics, and abilities to preserve the rounded cell shape that is essential for promoting the chondrogenic phenotype [73,74,75,76]. Thus, HA and AL can be combined because HA promotes cellular activity, while AL provides the appropriate rheological properties for the bioprintability of the bioink.

Regarding the supporting biomaterials, thermoplastic polymers such as polylactic acid (PLA) are some of the most promising biomaterials in CTE [77]. PLA provides excellent mechanical properties, thermoplastic processability, and biological properties, such as biocompatibility and biodegradability, which are all strongly influenced by its stereochemistry and molecular weight [77]. PLA also has excellent bioresorption capabilities, allowing the integration of the polymer with host cells and tissues [78]. Upon contact with biological media, PLA is degraded by simply hydrolyzing the ester bond into lactic acid, which is converted into pyruvate in the tricarboxylic acid cycle and excreted in the form of carbon dioxide and water. These degradation products are either metabolized intracellularly or excreted through urine and breath [79]. This process is primarily influenced by the characteristics of the polymer such as crystallinity, molecular weight, etc., and the conditions of hydrolysis, including pH and temperature [80]. Numerous studies have demonstrated that PLA, when used as a scaffold in tissue engineering, supports cell proliferation and growth [81,82,83]; also, its low price makes it an ideal choice for use in 3D bioprinting [84,85,86]. Its use in biomedical applications has been approved by the main health agencies such as the Federal Drug Administration (FDA, USA) and the European Medicine Agency (EMA, European Union) [87]. Moreover, PLA has been reported to be an effective scaffold agent alone or in combination with hydrogels [84,88].

Furthermore, for the bioprinting of biomimetic hybrid construct, the selection of a cell type to combine with the hydrogels is crucial to formulate suitable bioinks. hMSCs are regarded as an attractive cell type for cartilage regeneration because they are multipotent stem cells, with a high capacity to self-renew and differentiate into various cell types, including cartilaginous lineage [89,90,91,92]. hMSCs have been used as therapeutic agents not only for their healing capacities performed through engraftment and differentiation but also through paracrine signaling. These cells are capable of secreting soluble factors, which are indispensable for the viability, proliferation, and differentiation of cells surrounding a defect, for hyaline cartilaginous ECM stimulation, and for modulating the immune response [93,94]. Moreover, hMSCs overcome the limitations of chondrocytes, which are the only highly specialized cells found in mature AC [95]. The main limitation of in vitro chondrocytes culture is the dedifferentiation that occurs during expansion in monolayer culture. Chondrocytes do not maintain their characteristic phenotype, and, as a result, their capacity to regenerate damaged cartilage is compromised [96]. Additionally, the scarcity of donor tissue presents another challenge to the use of autologous chondrocytes [12]. Among the sources of hMSCs, adipose tissue presents several advantages in comparison to other tissues (i.e., bone marrow), including easier access, less invasiveness [97], and a greater cell yield per unit of tissue as well [98]. Specifically, Hoffa’s fat pad tissue has been demonstrated to contain multipotent and highly clonogenic adipose-derived stem cells [99,100].

To date, although much advancement in CTE strategies using 3D bioprinting systems have been reported, the bioprinting of a construct composed of a component of AC ECM, such as HA [101,102,103,104], with appropriate cell and structural properties that lead to hyaline cartilage tissue generation is still a challenge. Accordingly, in this study, we successfully developed a cartilage-like biomimetic hybrid construct (CBC) by 3D bioprinting as an effective approach to treat and support the regeneration of AC defects and to be applied affordably in the clinical area. The bioprinting of CBCs was performed using bioink, formulated with HA and AL and loaded with hMSCs, which were simultaneously bioprinted with PLA. Then, the CBC was crosslinked post-bioprinting with calcium chloride, obtaining a novel tissue-engineered product, which mimicked the environment and structural properties of native AC. The functionality of the CBCs was evaluated by testing their cell viability, proliferation, and chondrocyte differentiation by protein and gene expression assays, comparing them with a control CBC based on hMSC-AL-bioink (a construct without HA). Moreover, the physicochemical properties of the designed CBCs such as their porosity, degradation, swelling, surface electrical properties, conductivity, and environmental scanning electron microscopy were assessed compared to cell-free CBCs.

## 2. Materials and Methods

### 2.1. Isolation and Culture of hMSCs

hMSCs were isolated from infrapatellar fat pad (IFP) of patients with osteoarthritis during joint replacement surgery. Ethical approval for the study was obtained from the Ethics Committee (number: 02/022010) of the Clinical University Hospital of Málaga, Spain. Informed patient consent was obtained for all samples used in this study. The fat tissue was minced and digested using an enzymatic solution of 1 mg/mL collagenase type IA (Sigma-Aldrich, St. Louis, MO, USA) and incubated on a shaker at 37 °C for 1 h. After digestion, collagenase was removed by a single wash in sterile phosphate-buffered saline (PBS; Sigma-Aldrich, St. Louis, MO, USA), followed by two additional washes in Dulbecco’s Modified Eagle Medium (DMEM; Sigma-Aldrich, St. Louis, MO, USA) supplemented with 10% fetal bovine serum (FBS; Invitrogen, Waltham, MA, USA). The cell pellet was resuspended in DMEM (Sigma-Aldrich, St. Louis, MO, USA) containing 10% FBS and 1% penicillin/streptomycin (Sigma-Aldrich, St. Louis, MO, USA), added to tissue culture flasks, and cultured at 37 °C in 5% CO_2_. At 80% of confluency, the cells were released with TriPLE (Invitrogen, Waltham, MA, USA) and subcultured. The medium was changed regularly every 3 days, and cells were used at passage 4 for all the experiments.

### 2.2. Preparation of hMSC-Loaded Bioinks

The hMSC-loaded AL/HA-bioink (hMSC-AL/HA-bioink) was developed by preparing a solution of AL (2% *w*/*v*; molecular weight of AL: 216.12 g/mol; Sigma) and HA (1% *w*/*v*; molecular weight: 1000 kDa; Bioiberica S.A.U., Barcelona, Spain) Both materials were sterilized by short cycle autoclaving prior to preparation. This techniques has been previously shown to be effective in the sterilization of materials such as HA against some types of bacteria without affecting rheology, physicochemical properties, and printability [105], and it has also been used for the sterilization of the AL in other previous works [40,76,106]. hMSCs were then suspended in 1 mL of AL/HA-bioink at the concentration of 1 × 10^6^ cells/mL, resulting in the hMSC-AL/HA-bioink, which was packed into syringes (3 cc). Additionally, two other bioinks were also formulated as controls: (i) an hMSC-AL-bioink without HA and (ii) an AL/HA-bioink without cells.

### 2.3. Biofabrication of Cartilage-like Biomimetic Hybrid Construct by 3D Bioprinting

**Figure 1:**
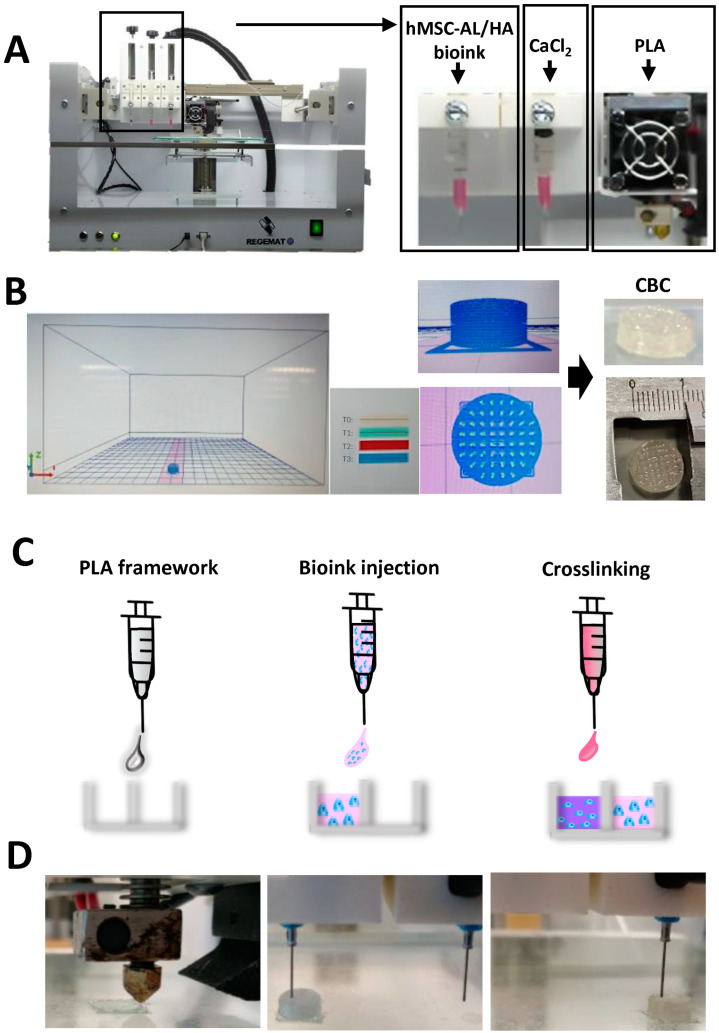
Three-dimensional bioprinting of CBCs based on hMSC-AL/HA-bioink. (**A**) REGEMAT Bioprinter system and bioinks. (**B**) Design of CBCs using REGEMAT software designer. (**C**) Scheme of dispensing and crosslinking of bioink in the PLA framework: hMSC-AL/HA-bioink (pink), calcium solution (blue), and crosslinked hMSC-AL/HA-bioink (purple). (**D**) Steps of biofabrication procedure of CBCs based on hMSC-AL/HA-bioink by 3D bioprinting.

The CBCs were bioprinted using REGEMAT V2 bioprinter (REG4Life, REGEMAT 3D, Granada, Spain) under aseptic conditions, as previously described [70] (Figure 1A). Briefly, PLA (Smart Materials 3D; tensile strength: 55.5 Mpa, specific gravity: 1.24 g/cm^3^, glass transition temperature: 60 °C, hardness: 85 shore D, diameter: 1.75 mm) was first deposited by head (at 200 °C) in a layer-by-layer manner to generate the framework, which was previously designed using software REGEMAT 3D designer v1.4.4 (porous cylinder-type structure, 5 mm high × 10 mm wide structure; 375 μm pore size) (Figure 1B,C). The selected pore size was based on previous studies of CTE (120–870 µm) [107]. After depositing 4 layers of PLA, the hMSC-AL/HA-bioink was injected between PLA strands obtaining the constructs, which were then physically crosslinked using a bath of 100 mM calcium chloride (CaCl_2_; Panreac, Barcelona, Spain), loaded into another syringe, for 30 min [108] (Figure 1C,D). In addition, two types of control constructs were bioprinted from hMSC-AL-bioink and AL/HA-bioink. It was biofabricated for as many constructs as needed for the following analytical studies.

### 2.4. CBC Maturation in Cell Culture

CBCs based on hMSC-AL/HA-bioink and control constructs based on hMSC-AL-bioink were cultured at 37 °C and 5% CO_2_ atmosphere with high-glucose DMEM (Sigma) supplemented with 10% FBS (Lonza, Norwest, NSW, Australia) and 1% penicillin/streptomycin (Sigma) for a maximum of 5 weeks.

### 2.5. Flow Cytometry Analysis

The immunophenotype of hMSCs was analyzed by flow cytometry using fluorescence-activated cell sorter (FACS). Cells were washed and resuspended in PBS (Sigma) with 2% bovine serum albumin (BSA, Sigma) and 2 mM ethylenediaminetetraacetic acid (EDTA; Sigma). Cells were incubated in the dark for 30 min at 4 °C with the appropriate fluorochrome-conjugated monoclonal antibodies. The markers used were CD73-APC, CD90-FITC, CD105-PE, CD45-PerCP, CD19-APC, and HLA-DR-FITC (Miltenyi Biotec, Macquarie Park, NSW, Australia). After incubation, cells were washed in PBS and analyzed in a FACS Canto II cytometer (BD Biosciences, Franklin Lakes, NJ, USA).

### 2.6. Cell Differentiation STAINING Assay

For adipogenic, osteogenic, and chondrogenic differentiation, hMSCs were cultured for two weeks in Adipogenic MSC Differentiation Bullet kit, Osteogenic MSC Differentiation Bullet kit (Lonza), and NH ChondroDiff Medium (Miltenyi), respectively. Differentiated cell cultures were stained with Oil Red O (Amresco, Solon, OH, USA) for adipogenic differentiation, Alizarin Red (Lonza) for osteogenic differentiation, or Toluidine Blue (Sigma) for chondrogenic differentiation and imaged using an optical microscope (Leica DM 5500B; Leica Microsystems, L’Hospitalet de Llobregat, Spain).

### 2.7. Cell Viability Assay

The live/dead kit assay (Thermo Fisher Scientific, Waltham, MA, USA) was used to evaluate cell viability of cells embedded in CBCs and control constructs based on hMSC-AL-bioink, following manufacturer’s instructions. Stained constructs from day 1, 3, 5, 7, 14, and 21 days in culture were observed using a confocal microscope (Nikon Eclipse Ti-E; Nikon Instruments Europe B.V., Amsterdam, The Netherlands) by two different filters. Green fluorescence was visualized in live cells and red fluorescence in dead cells. Confocal images were analyzed with Image J software (v. 1.52i, National Institutes of Health, Bethesda, MD, USA). The percentage of viable cells were obtained by counting six regions of each sample (n = 3).

### 2.8. Cell Proliferation

The proliferation rate of cells (n = 3) into CBCs and control constructs based on hMSC-AL-bioink were assessed by colorimetric Alamar Blue (aB) assay (Thermo Fisher Scientific) at initial and final culture time (day 0 and day 21, respectively), following manufacturer’s instructions. Fluorescence intensity was measured with an excitation wave length of 530 nm and emission of 590 nm using a spectrophotometer (Synergy HT, BIOTEK, San Diego, CA, USA). The absorbance data were represented as fold increase to initial culture time.

### 2.9. Biochemical Assay

CBCs and control constructs based on hMSC-AL-bioink were digested in 1 mL of papain solution (125 mg/mL papain in 0.1 M sodium phosphate with 5 mM Na_2_ EDTA and 5 mM cysteine HCl at pH 6.5) for 16 h at 60 °C, followed by centrifugation at 10,000 rpm for 5 min. The supernatant was used for chemical assay. The amount of GAGs was measured by the dimethylmethylene blue (DMMB) colorimetric assay. The supernatant was mixed with DMMB solution to bind GAGs. The GAG content was calculated based on a standard curve of chondroitin sulfate from shark cartilage (Sigma) at 530 nm using a microplate spectrophotometer and normalized with cell-free hybrid constructs. The DNA content was determined by a Hoechst assay and calculated using thymus DNA for a standard curve (Sigma). The supernatant was reacted with the Hoechst dye for 30 min in the dark. The intensity of fluorescence was screened with a 96-well plate reader (excitation at 360 nm and emission at 460 nm).

For type II collagen quantification, samples were digested by pepsin (1 mg/mL) in 0.5 N acetic acid for 48 h at 4 °C, followed by adding 1 mg/mL pancreatic elastase solution at 4 °C for 24 h. Finally, the samples were neutralized with 1 M Tris base. Insoluble material was removed by centrifugation at 10,000 rpm at room temperature for 5 min, and the supernatant was collected for assay. Quantitative analysis was performed using a commercially available type II collagen ELISA kit (Chondrex, Woodinville, WA, USA), according to manufacturer’s instruction and measured on microplate spectrophotometer at 490 nm.

### 2.10. RT-PCR Analysis

Total messenger RNA (mRNA) of cells in CBCs and control constructs based on hMSC-AL-bioink (a construct without HA) was isolated using TriReagent (Sigma) and reverse transcribed into cDNA using the Reverse Transcription System kit (Promega, Madison, WI, USA). The quantitative real-time polymerase chain reaction (qRT-PCR) was conducted using a SYBR green master mix (Promega) according to the manufacturer’s recommendations. Gene expression levels for aggrecan (*ACAN*), type II collagen (*COL2A1*), Sox transcription factor 9 (*SOX9*), type X collagen (*COL10A1*), and the RUNX2 gene (*RUNX2*) of the CBCs were normalized to the housekeeping gene glyceraldehyde 3-phosphate dehydrogenase (GAPDPH) and showed a fold change relative to the value of the control constructs. The primer sequences are reported in a previous work [109]. All the samples were analyzed in triplicate for each gene.

### 2.11. Physicochemical Characterization

#### 2.11.1. Porosity Analysis

The porosity of CBCs based on hMSC-AL/HA-bioink and cell-free CBCs based on AL/HA-bioink was determined by the solvent replacement method. Briefly, the dry samples were submerged in 20 mL absolute ethanol and then weighed after removing the excess of ethanol with tissue paper. The porosity of samples was obtained by the following equation [110,111]:

(1) $$Porosity=\frac{W2-W1}{\rho \times V}\times 100$$

where W2 is the weight of ethanol-absorbed samples, W1 is the weight of dry samples, ρ is the ethanol density, and V is the volume of samples.

#### 2.11.2. Swelling Test

The water uptake capacity of the CBCs based on hMSC-AL/HA-bioink and cell-free CBCs based on AL/HA-bioink was determined by swelling dried sample (with known weights) in 15 mL PBS (pH 7.4) at 37 °C. At predetermined intervals, samples were taken out, surface adsorbed water was removed by filter paper, and the wet weight was recorded. Each measure was carried out in triplicate. The swelling ratio was calculated as follows [112]:

(2) $$Swelling\;ratio\left(\%\right)=\frac{Ws-Wd}{Wd}\times 100$$

where Ws is the weight of the samples at the swelling state, and Wd is the initial mass of dried samples.

#### 2.11.3. In Vitro Degradation Analysis

Before beginning in vitro degradation, CBCs based on hMSC-AL/HA-bioink and cell-free CBCs based on AL/HA-bioink were pre-wetted by immersion in PBS (pH = 7.4 ± 0.05) at 37 °C. Once equilibrium was reached, samples were weighed and recorded as W0. Samples were then incubated in 15 mL PBS at 37 °C. At specific time intervals, samples were removed and dried in a vacuum oven at 50 °C for 60 min, and their dry weight was recorded. The samples were then returned to containers containing PBS [113]. The degradation (%) was calculated in triplicate according to the following equation [114]:

(3) $$Degradation\;ratio\left(\%\right)=\frac{W0-Wt}{W0}\times 100$$

where W0 is the initial mass of sample, and Wt is the mass of degraded sample measured at time t after drying at 50 °C in vacuum oven for 60 min.

#### 2.11.4. Surface Electrical Properties 

Sample preparation for zeta potential (ζ) and conductivity analysis of CBCs based on hMSC-AL/HA-bioink and cell-free CBCs based on AL/HA-bioink was performed as previously reported [43]. Briefly, samples were dried (10 mg dried mass) at 50 °C for 24 h. Then, they were suspended in double-distilled water and sonicated for 1 h at 50% frequency for further analysis. ζ was determined by electrophoresis measurements using a Zetasizer Nano ZS (Malvern Instruments Ltd., Malvern, UK). Furthermore, the surface electrical properties of samples as a function of pH variation (from 4 to 8, at constant 10^−3^ M KNO_3_ concentration) and ionic strength (from 10^−1^ to 10^−5^ M KNO_3_, at pH = 6) were studied. Values are reported as the mean ± SD (n = 9).

On the other hand, electrical conductivity of CBCs based on hMSC-AL/HA-bioink and cell-free CBCs based on AL/HA-bioink was measured with a conductometer Crison EC-Meter BASIC 30+ (Crison Instruments, Alella, Barcelona, Spain). An electrode was dipped into a glass vial of sample. Data were expressed as mean ± SD (n = 3).

### 2.12. Scanning Electronic Microscopy (SEM)

To analyze internal microstructure of CBCs based on hMSC-AL/HA-bioink and cell-free CBCs based on AL/HA-bioink, the samples were fixed in cold 2.5% glutaraldehyde and rinsed in PBS, followed by a dehydration process through a graded series of ethanol (30–100%), and finally critically point-dried in an Emscope CPD 750 critical point dryer. Hybrid constructs were attached to aluminum SEM specimen mounting stubs (Electron Microscopy Sciences, Hatfield, PA, USA) and sputter-coated with a gold palladium alloy using a Sputter Coater 108 Auto. Finally, samples were examined using a scanning electron microscope eSEM (FEI Quanta 400, OR, USA)and a field emission scanning electron microscope JSM-7001F (JEOL Ltd., Tokyo, Japan). Images were taken at several magnifications.

### 2.13. Mechanical Testing of the Scaffolds

The compression modulus was measured using a universal testing machine (AGS-X Shimadzu, Kyoto, Japan) at a constant approaching speed from 0 to 0.1 mm. Measurements were conducted under controlled temperature and humidity conditions to minimize potential variations in material properties (n = 5).

### 2.14. Statistical Analysis

All data were represented as mean ± SD. The two-tailed Student’s *t*-test was used to determine differences between conditions. *p*-values < 0.01 (**) and <0.05 (*) were considered statistically significant in all cases.

## 3. Results

### 3.1. CBC Biofabrication

The biofabrication of CBCs was based on a co-deposition of bioink formulated from HA and AL loaded with hMSCs mimicking the microenvironment and composition of the ECM of native AC with a PLA framework used as the mechanical support of the structure. CBCs based on hMSC-AL/HA-bioink, control constructs based on hMSC-AL-bioink, and cell-free CBCs based on AL/HA-bioink were successfully bioprinted by the combination of two procedures, Fused Deposition Model (FDM) and Injection Volume Filling (IVF), which are incorporated in the REGEMAT 3D V2 system. Each bioink was injected between PLA frames in a layer-by-layer manner, resulting in a 5 mm high × 10 mm wide structure. Then, constructs were immediately crosslinked with a calcium chloride bath (Figure 1C).

### 3.2. Flow Cytometry Analysis

**Figure 2:**
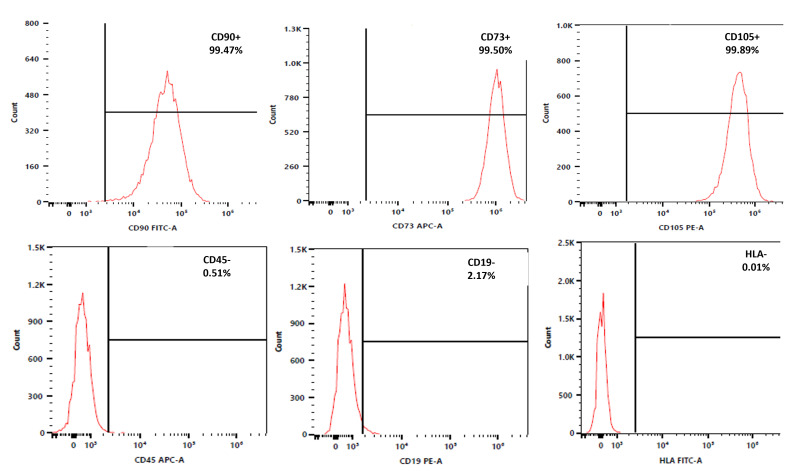
Phenotypic characterization of hMSCs. FACS characterization of hMSCs showed a positive expression of the surface markers CD90, CD73, and CD105 and negative or low expression of CD45, CD19, and HLA.

Following the recommendation of the International Society for Cellular Therapy (ISCT), the hMSCs were characterized according to the minimal criteria for defining multipotent mesenchymal stromal cells. Flow cytometry analysis of the hMSCs revealed a high expression of CD90 (99.47%), CD73 (99.5%), and CD105 (99.89%) markers while showing a negative or low expression of CD45 (0.51%), CD19 (2.17%), and HLA (0.01%) markers (Figure 2).

### 3.3. Cell Differentiation Staining Assay

**Figure 3:**
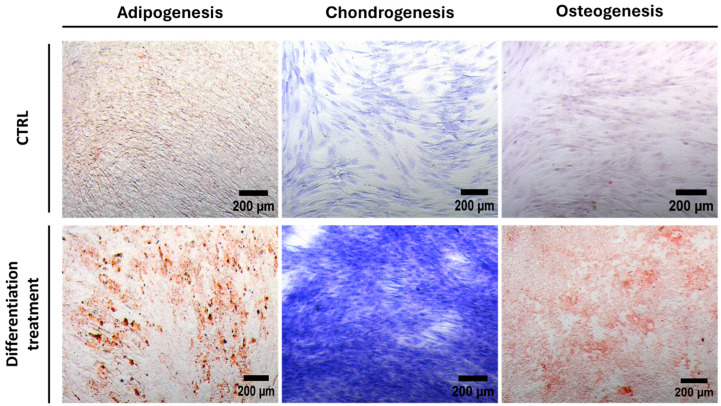
Cell differentiation staining assay. The differentiation potential of hMSCs obtained from IFP towards osteogenic, adipogenic, and chondrogenic lineage was confirmed by Alizarin red S, Oil Red O, and Toluidine Blue staining, respectively. Scale bar: 200 μm. Images were taken at 10× magnification.

The plasticity potential of the isolated cells was assessed to evaluate their ability to differentiate into chondrocytes, adipocytes, and osteoblasts. hMSCs were cultured under standard in vitro differentiating conditions for two weeks. Adipogenic differentiation was confirmed by the presence of lipid deposits stained with Oil Red. Osteogenic differentiation was determined by calcific deposition using Alizarin Red S staining. Chondrogenic differentiation was assessed by the presence of proteoglycans stained with Toluidine Blue. Thus, cells isolated from the IFP demonstrated the ability to differentiate into various mesenchymal tissue types, including adipocytes, chondrocytes, and osteoblasts (Figure 3).

### 3.4. Cell Viability

**Figure 4:**
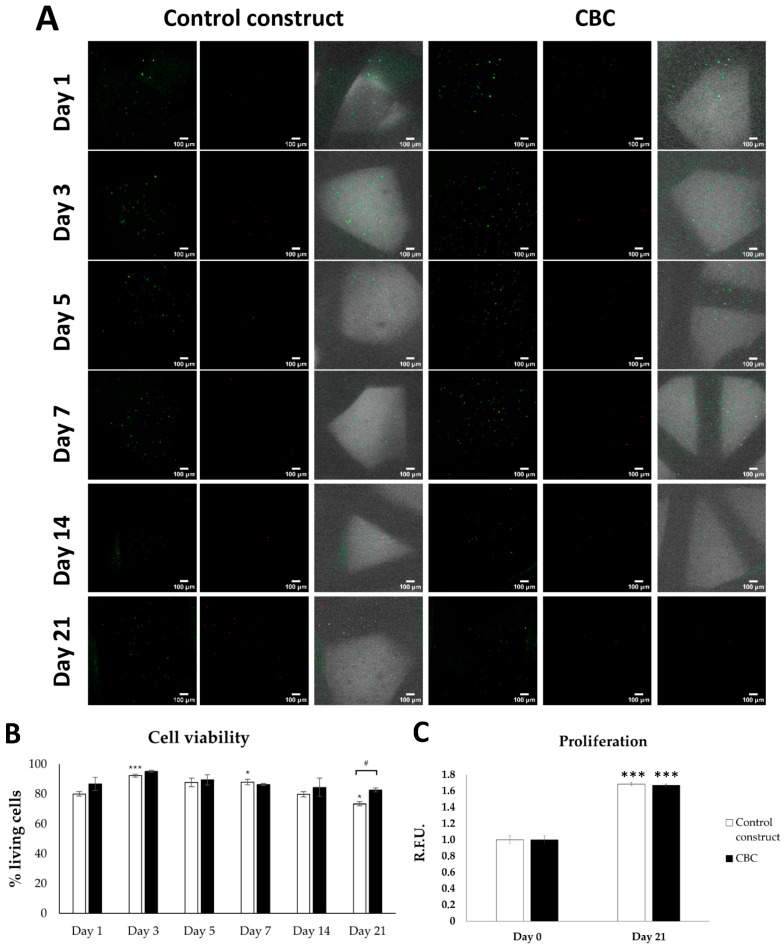
Cell viability and proliferation of hMSCs in control constructs based on hMSC-AL-bioink and CBCs based on hMSC-AL/HA-bioink. (**A**) Representative image of hMSCs in control constructs and CBCs after 1, 3, 5, 7, 14, and 21 days in culture, showing live (green) and dead (red) cells. (**B**) Percentage of hMSC viability in control constructs and CBCs with respect to time in culture. (**C**) Cell proliferation inside the CBCs based on hMSC-AL/HA-bioink (black) and control constructs based on hMSC-loaded AL-bioink (white). Error bars represent standard deviations (n = 3). (*) *p* < 0.05; (***) *p* < 0.005; (#) *p* < 0.05. Scale bar: 100 μm.

The viability of hMSCs in the CBC based on hMSC-AL/HA-bioink and in the control constructs based on hMSC-AL-bioink were analyzed at day 1, 3, 5, 7, 14, and 21 using life/dead staining assay. Confocal images showed a uniform distribution of cells within the CBC and in the control construct for the entire study period. hMSCs were embedded throughout the AL and AL/HA bioinks between the PLA strands (Figure 4A), being the majority viable cells (green-stained cells). After the bioprinting process (day 1), around 90% of living cells in the CBC and more than 80% of living cells in the control construct were observed. Long-term analysis also indicated that cell viability remained high (>85%) throughout the 14 days in the culture, slightly increasing from days 1 to 3 (Figure 4B). There is a significant difference in the control group between days 3, 7, and 21 compared to day 1. Additionally, significant differences were observed between the control construct group and the CBC groups, but only at day 21.

### 3.5. Cell Proliferation

Cell proliferation in CBCs based on hMSC-AL/HA-bioink and control constructs based on hMSC-AL-bioink was analyzed at initial and final time points of culture. Figure 4C shows that the proliferation rate of cells in CBCs is equal or comparable to the control constructs at both 0 and 21 days. After 21 days, cells exhibited an increase in growth in comparison to day 0. Although there were no significant differences between the control constructs and CBCs, significant differences were observed within each group over time, i.e., the metabolic activity of the cells increases over time for both conditions.

### 3.6. Chondrogenesis of hMSCs in CBCs

**Figure 5:**
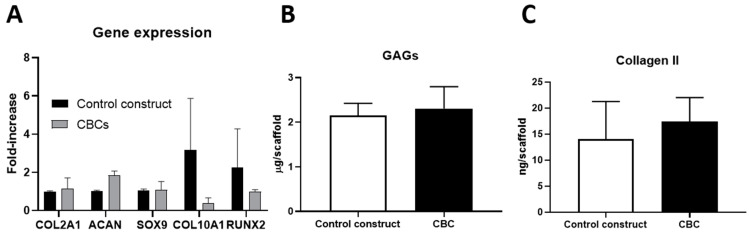
Chondrogenesis of hMSCs in CBCs based on hMSC-AL/HA-bioink and in control constructs based on hMSC-AL-bioink after 5 weeks in culture. (**A**) Gene expression levels of hyaline-specific chondrogenic marker genes (*COL2A1*, *ACAN*, and *SOX9*) and other genes such as *COL10A1* and *RUNX2* from cells in CBCs based on hMSC-AL/HA-bioink compared to cells of control constructs. (**B**) Quantitative analysis of GAGs. (**C**) Quantitative analysis type II collagen in the total extract per construct. Values represent mean ± SD (n = 3).

The capacity of hMSC-AL/AH-bioink to enhance chondrogenic differentiation and, therefore, cartilage formation in hybrid constructs was evaluated. Figure 5A shows the gene expression of hMSCs in CBC based on hMSC-AL/HA-bioink, after 5 weeks in culture, compared to hMSCs in control constructs based on hMSC-AL-bioink (without HA). For that, *COL2A1*, *ACAN*, *SOX9*, *COL10A1*, and *RUNX2* genes were analyzed. Results from qPCR analysis revealed an increase in mRNA levels of hyaline cartilage-specific genes such as *COL2A1*, *ACAN*, and *SOX9* from hMSCs bioprinted in CBCs based on hMSC-AL/HA-bioink in comparison to those bioprinted in the control group based on hMSC-AL-bioink, while the expression of the hypertrophic marker gene *COL10A1* was decreased in CBCs. The results for RUNX2 expression (an essential transcription factor for osteoblast differentiation) showed a slight decrease in CBCs. No significant differences were observed in the expression of any of the genes (*p* > 0.05).

hMSC chondrogenic differentiation was also analyzed at the protein level by measuring the production of the main components of chondrogenic matrix (GAGs and collagen II) after 5 weeks in culture. Results from biochemical assays indicated that GAG content in CBC based on hMSC-AL/HA-bioink (2.31 µg/construct) was slightly higher than those in the control construct based on hMSC-AL-bioink (2.15 µg/construct). Similarly, the amount of type II collagen was also higher in CBC based on hMSC-AL/HA-bioink (17.4 ng/construct) compared to the control construct (14.0 ng/construct), but, in both cases, such difference was not significant (*p* > 0.05) (Figure 5B,C).

### 3.7. Physicochemical Characterization of CBCs

The physicochemical characterizations of CBCs based on hMSC-AL/HA-bioink and cell-free CBCs based on AL/HA-bioink were conducted to evaluate whether the addition of cells modified the characteristics of the CBCs.

#### 3.7.1. Porosity

Porosity is an important characteristic of constructs for TE, as it influences degradation, swelling capacity, and mechanical properties. The porosity of CBCs based on hMSC-AL/HA-bioink was higher than cell-free CBCs based on AL/HA-bioink, measuring 18 ± 2.4% and 2 ± 0.9%, respectively.

#### 3.7.2. Swelling Test

**Figure 6:**
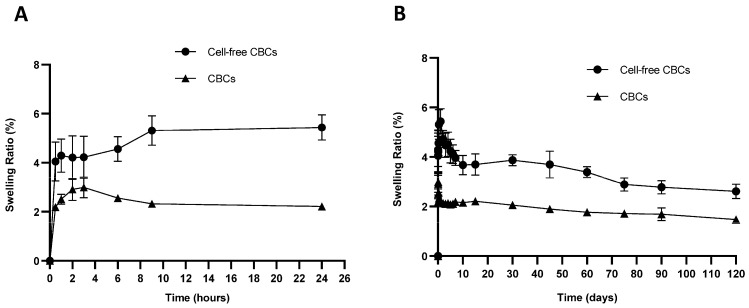
Swelling ratio of CBCs based on hMSC-AL/HA-bioink and cell-free CBCs based on AL/HA-bioink. (**A**) Swelling ratio at pH 7.4 and 37 °C after 24 h. (**B**) Swelling ratio at pH 7.4 and 37 °C after 120 days. Values represent mean ± SD (n = 3).

The water absorption ability was evaluated by immersing the samples in PBS at 37 °C for 120 days. Figure 6 shows the swelling behavior of CBCs based on hMSC-AL/HA-bioink and cell-free CBCs based on AL/HA-bioink. Low rates of swelling in both CBCs based on hMSC-AL/HA-bioink and cell-free constructs can be seen. The maximum swelling rate of CBCs based on hMSC-AL/HA-bioink was reached after 3 h, with a swelling percentage of 3.0 ± 0.4%, whereas the cell-free constructs reached their maximum swelling after 24 h, with a swelling percentage of 5.4 ± 0.5%. Afterward, the samples started to lose weight, and their swelling ratio seemed to stabilize with time.

#### 3.7.3. In Vitro Degradation Analysis

**Figure 7:**
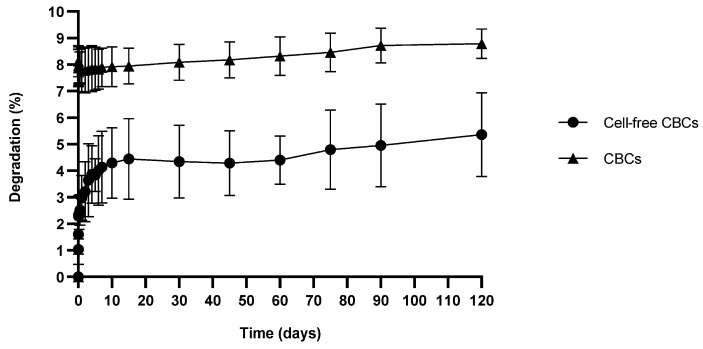
Degradation percentage of CBCs based on hMSC-AL/HA-bioink and cell-free CBCs based on AL/HA-bioink at pH 7.4 and 37 °C. Values represent mean ± SD (n = 3).

The degradation of CBCs based on hMSC-AL/HA-bioink and cell-free CBCs based on AL/HA-bioink was expressed as the percentage of weight loss (Figure 7). This was evaluated by measuring the weight change of the samples after immersion in PBS at 37 °C for 120 days. The results indicate low percentages of degradation for both CBCs based on hMSC-AL/HA-bioink and cell-free CBCs based on AL/HA-bioink after 120 days of incubation. The degradation profile was higher for CBCs based on hMSC-AL/HA-bioink, with a weight loss of 8.8 ± 0.6%, compared to 5.4 ± 1.6% for the cell-free CBCs.

#### 3.7.4. Surface Electrical Properties and Conductivity Analysis

**Figure 8:**
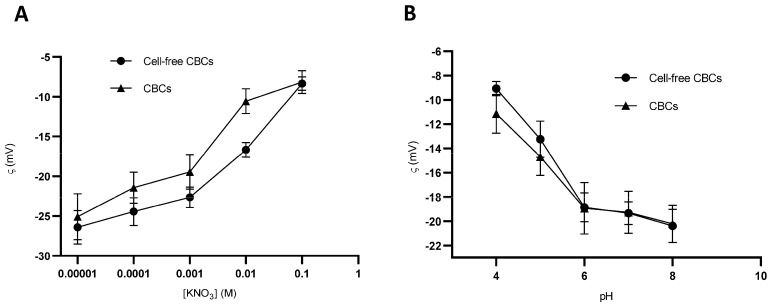
Zeta potential (ζ, mV) of CBCs based on hMSC-AL/HA-bioink and cell-free CBCs based on AL/HA-bioink. (**A**) ζ as a function of ionic strength at pH = 6 at 25 °C. (**B**) ζ as a function of pH in the presence of 10^−3^ M KNO_3_ concentration at 25 °C. Values represent mean ± SD (n = 9).

The electrophoretic characterization of CBCs based on hMSC-AL/HA-bioink and cell-free CBCs based on AL/HA-bioink is depicted in Figure 8. It can be observed a negative ζ ionic strength range both in CBCs based on hMSC-AL/HA-bioink and cell-free CBCs based on AL/HA-bioink (Figure 8A). The ζ showed a dependence on the ionic strength, with a slight decrease in negative ζ as KNO_3_ concentration increased in both cases. Results of ζ ranged from −8.2 ± 1.4 mV to −25.1 ± 2.9 mV and −8.3 ± 0.8 mV to −26.4 ± 2.1 mV for CBCs based on hMSC-AL/HA-bioink and cell-free CBCs based on AL/HA-bioink, respectively. On the other hand, Figure 8B shows the ζ values at different pHs ranging from 4 to 8 in the presence of 10^−3^ M KNO_3_ concentration at 25 °C. A negative ζ was maintained across the pH range for both CBCs based on hMSC-AL/HA-bioink and cell-free CBCs based on AL/HA-bioink. The ζ (in an absolute value) followed an increase as pH increased from −11.2 ± 1.6 mV to −20.2 ± 1.5 mV for CBCs based on hMSC-AL/HA-bioink and from −9.1 ± 0.6 mV to −20.4 ± 1.4 mV for cell-free CBCs based on AL/HA-bioink.

Finally, the conductivity values for CBCs based on hMSC-AL/HA-bioink and cell-free CBCs based on AL/HA-bioink were 160.0 ± 2.0 µS/cm and 75.7 ± 5.0 µS/cm, respectively. These results indicated that the incorporation of hMSCs can greatly improve the conductivity of CBCs.

### 3.8. SEM Microscopy Analysis

**Figure 9:**
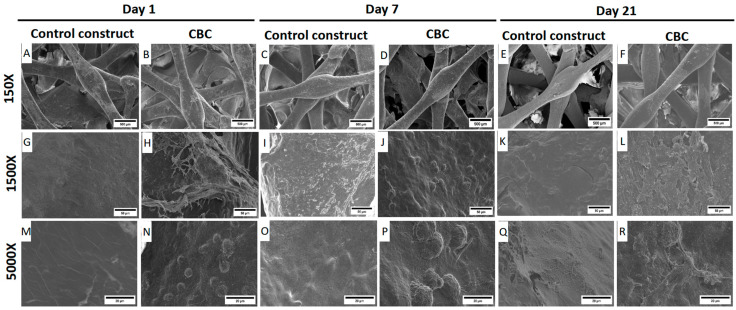
SEM microscopy analysis of hMSCs in constructs. (**A**,**G**,**M**) Cell-free CBCs based on AL/HA-bioink in cell culture conditions after 1 day. (**B**,**H**,**N**) CBCs based on hMSC-AL/HA-bioink in cell culture conditions after 1 day. (**C**,**I**,**O**) Cell-free CBCs based on AL/HA-bioink in cell culture conditions after 7 days. (**D**,**J**,**P**) CBCs based on hMSC-AL/HA-bioink in cell culture conditions after 7 days. (**E**,**K**,**Q**) Cell-free CBCs based on AL/HA-bioink in cell culture conditions after 21 days. (**F**,**L**,**R**) CBCs based on hMSC-AL/HA-bioink in cell culture conditions after 21 days. Images were taken at 150× (**A**–**F**; scale bar: 500 μm), 1500× (**G**–**L**; scale bar: 50 μm), and 5000× (**M**–**R**; scale bar: 20 μm).

The microstructure of CBCs based on hMSC-AL/HA-bioink and cell-free CBCs was analyzed by SEM. Images were taken on day 0, 7, and 21 with the optical microscope. Figure 9 shows the internal distribution of the main components in bioprinted constructs. SEM images of CBC based on hMSC-AL/HA-bioink revealed the presence of hMSCs, while no cells are visible in cell-free CBCs. The hydrogel within the constructs is clearly observable in the 150× magnification images. Over time, the micrographs showed improved cell adhesion and cell growth in the constructs (Figure 9N,P,R).

### 3.9. Mechanical Test

The mechanical properties of scaffolds are crucial in the development of an effective artificial substitute. The ability of a construct to withstand deformation under an applied load is known as elastic or Young’s modulus (E). The result obtained in this test shows that the CBCs have a Young’s modulus of 4.069 ± 0.567 MPa. This result indicates that the CBCs possess adequate stiffness to potentially function effectively as a scaffold in tissue-engineering applications.

## 4. Discussion

Three-dimensional bioprinting technology offers a therapeutic alternative in the treatment of cartilage injuries [115]. The bioprinting of biomimetic hybrid constructs through the controlled and simultaneous deposition of hydrogels, as cell carriers, and thermoplastic polymers, as scaffolding biomaterials, has been demonstrated to be the most suitable strategy for cartilage regeneration. The selection of suitable biomaterials for bioprinting a construct is essential, especially for the formulation of cell-laden bioinks. Bioinks are mainly formulated from hydrogels, which not only must provide a good biomimetic environment for cells to grow and differentiate correctly but also must protect them from damaging conditions generated during the 3D bioprinting process, such as heat and shear stress [34,116,117], ensuring cell viability within the construct at the time of its implantation. Supportive biomaterials must also have good biodegradability and appropriate porosity to ensure cellular penetration and the adequate diffusion of nutrients to cells. In addition, a construct should provide mechanical properties consistent with the damage tissue to treat [118,119]. Tissue bioprinting presents several challenges for its translation into clinical practice, primarily in sourcing patient-derived cells to minimize immune rejection. This process involves complexities in cell collection and expansion and in ensuring adequate cell counts for successful bioprinting and tissue maturation. Additionally, ensuring compatibility for long-term storage and addressing sourcing and batch variability of bioinks are critical issues. Advancements in bioprinting techniques and the standardization of processes are essential for scalable production. Ethical, legal, and social considerations surrounding bioprinted tissues also need thorough deliberation [120,121].

In this study, a CBC was developed from hMSC-AL/HA-bioink. This bioink combined HA with AL, which is considered one of the most appropriate biomaterials for bioprinting [31,122,123]. The formulation of the hMSC-AL/HA-bioink, composed of 1% HA and 2% AL, was selected based on our previous study in which it was demonstrated to better promote chondrogenic phenotype and hyaline-cartilage-specific matrix formation in comparison to other combinations [70].

For that, we successfully developed a novel CBC by 3D bioprinting to facilitate and promote cartilage regeneration. The CBCs were biofabricated by co-printing the hMSC-AL/HA-bioink with a supporting thermoplastic biomaterial, such as PLA, to mimic both the environment and mechanical properties of native AC. In our previous study, we demonstrated that the mixture of HA with AL has excellent biological and rheological properties to be used as cell-carrier biomaterial for CTE bioprinting, including a cartilage-like environment, printability, and gelling abilities [70]. In term of cells, hMSCs from adipose tissue represent an ideal cell source to treat cartilage defects, not only due to their potential to differentiate into cartilage lineage but also due to their easy accessibility and abundance in the body, overcoming the limitations associated with chondrocytes [124].

The ISCT proposed minimal criteria to define hMSCs. First, hMSCs must be plastic-adherent when maintained in standard culture conditions. Second, hMSC must express CD105, CD73, and CD90 and lack the expression of CD45, CD34, CD14, CD11b, CD79α, or CD19 and HLA-DR surface molecules. Third, hMSCs must differentiate into osteoblasts, adipocytes, and chondroblasts in vitro [125,126]. The fluorescence-activated cell sorting analysis of hMSCs showed a positive expression of the surface markers CD90, CD73, and CD105 and a negative expression of CD45, CD19, and HLA-DR. We also tested the plasticity potential of the isolated cells by examining their ability to differentiate into chondrocytes, adipocytes, and osteoblasts using Alizarin Red S, Oil Red O, and Toluidine Blue staining. Cells isolated from the IFP of patients with osteoarthritis were able to differentiate into adipocytes, chondrocytes, and osteoblasts. These results confirmed the minimal phenotypic pattern for the identification of hMSC cells and the differentiation potential of hMSCs. Representative images for the live/dead assay of CBCs based on hMSC-AL/HA-bioink clearly indicated that cells were viable, emphasizing the potential of hMSC-loaded bioink as a cell encapsulating hydrogel for the biofabrication of cartilage constructs. In accordance with our previous work, this fact could be explained through the shear thinning behavior described previously, which reduces shear stress causative of cell disruption [70]. The results confirmed that hMSC-AL/HA-bioink offered a cell-friendly environment, maintaining a high percentage of viability in CBC based on hMSC-AL/HA-bioink over 21 days in culture, as has been well documented in many studies [127]. In addition to rheological properties of hMSC-loaded AL/HA-bioink, this high cell viability would be attributed in part to the microstructure of the bioprinted construct. By the analysis of cell proliferation, a significant increase in cell growth over this period (21 days) in CBC based on hMSC-AL/HA-bioink in comparison to control constructs based on hMSC-loaded AL-bioink was also evidenced. These results are in accordance with other studies that reported the beneficial effect of HA on cell proliferation [59,128,129]. In terms of functionality, it was observed that the environment provided by hMSC-loaded AL/AH-bioink in CBC is influenced by the commitment of hMSCs to undergo chondrogenesis. Previous studies have demonstrated that AL can induce chondrogenic differentiation and promote the retention of the spheroidal shape of cells, which is the differentiated phenotype of chondrocytes [76,90,130]. Meanwhile, HA has been shown to independently induce chondrogenesis on hMSCs [131] and to drive chondrocyte regeneration when it was used as injected scaffold [14,132,133]. In this study, the addition of HA to AL has demonstrated a positive effect, improving the ability of hMSCs to differentiate and produce the main components of chondrogenic ECM (GAGs and collagen II). The analysis of gene expression showed an increase in the expression of mature chondrocyte genes (*COL2A1*, *ACAN*, and *SOX9*) in the presence of HA. In accordance with our results, a stimulatory effect on the synthesis of collagen II and GAGs has been observed through various mechanisms. This effect is expected to result from cell receptor binding with HA (i.e., CD44, hyaluronan-mediated motility receptor). Chondrocytes and hMSCs have membrane receptors capable of binding to HA and, thus, being influenced by its presence [56]. Moreover, there was a trend towards a reduction in hypertrophic marker (COL10A1) and osteogenic marker (RUNX2). Type X collagen is located in hypertrophic cartilage and the calcified zone of articular cartilage. Hypertrophic chondrocytes directly promote changes of their surrounding matrix, which increases mineralization and reduces the elastic properties of the cartilage [134]. The low expression of COL10A1 observed suggests that the hMSCs are not differentiating towards a hypertrophic chondrocyte lineage, thereby reducing the risk that the regenerated cartilage will progress towards an undesirable phenotype that could compromise tissue functionality. Additionally, RUNX2 is a transcription factor of osteoblast differentiation; it plays a determinant role in the early osteogenic differentiation and has become a marker of early osteogenic differentiation [135]. The low expression of RUNX2 suggests that the cells are not progressing toward osteoblastic differentiation.

The knowledge of CBCs based on hMSC-AL/HA-bioink in comparison with the cell-free CBCs based on AL/HA-bioink is essential to predict if the presence of living cells inside the constructs can modify or alter their physicochemical characteristics [136]. The porosity, pore size, and pore-to-pore interconnectivity can facilitate the cellular migration and infiltration as well as the right flow nutrients and oxygen within the construct and metabolic waste disposal, creating a favorable environment for tissue regeneration [137,138,139,140]. In fact, our results showed that the addition of hMSCs led to an increase in porosity value, probably due to the spatial incorporation of the cells within the CBCs. These data were verified in SEM images, which revealed that the CBC based on hMSC-AL/HA-bioink, as well as the PLA framework that it contains, had enough large interconnected pores to allow the diffusion of nutrients, oxygen, and metabolism products within the cartilage matrix, which is essential for cell living and their growth. The porosity values of the PLA framework are in the range of values reported in previous studies about 3D scaffolds for CTE [107]. The SEM images showed the internal distribution of the main components in the bioprinted constructs and revealed the presence of hMSCs in the CBCs based on hMSC-AL/HA-bioink. After 21 days, CBCs based on hMSC-AL/HA-bioink improved cell adhesion and cell growth.

The ability to absorb liquid, or swelling, is an important property of constructs, as it is associated with the flow nutrients and oxygen through the material structure [141,142,143]. The low swelling data obtained may be due to a characteristic of PLA, which is a relatively hydrophobic thermoplastic polyester [144]. Cheng et al. also reported a low swelling ratio of the PLA scaffolds [145]. Despite this, the low swelling data did not compromise the cellular viability and probably contributed to prolonging the degradation process. Our results are similar to those obtained by Correira et al. They prepared freeze-dried composite scaffolds of chitosan and HA and showed low swelling values throughout the entire experiment. In addition, they demonstrated that the ratio between chitosan and HA significantly affected the scaffolds’ swelling properties. Despite these results, they suggested that the developed scaffolds could have potential use in the regeneration of cartilaginous lesions [141].

The degradation rate of a construct is a critical element for the repair process. The ideal construct should be biodegradable and allows for remodeling as the new cartilage forms, substituting the damaged cartilage. The construct should exhibit progressive degradation that coincides with ECM deposition and accumulation and align with the restoration of new tissue or function [138,141]. In our study, the degradation showed low rates in the developed CBCs. This slow degradation rate may be a consequence of the poor biodegradability of PLA, as demonstrated by Nazeer et al. and Cheng et al. in their research [145,146]. PLA degrades through the hydrolysis of backbone ester groups, which is enhanced by enzymes and depends on the ability of water to diffusion into the polymer [144,147]. During hydrolysis, PLA is broken down into water-soluble monomers and oligomers, which are eventually decomposed into water and carbon dioxide and removed. This process is mainly influenced by the characteristics of PLA (such as crystallinity, molecular weight, monomer concentration, conformation, and porosity [80,148], as well as the hydrolysis environment, including pH, temperature, and other factors. To accelerate the degradation of PLA, methods such as blending, compounding, copolymerization, and surface modification have been studied [80]. Apart from that, the degradation rate of AL-based biomaterials is also affected by the molecular weight of AL. Essentially, a higher molecular weight reduces the number of reactive sites accessible for hydrolytic degradation, leading to a slower degradation rate [149]. On the other hand, the presence of hMSCs rendered CBCs more susceptible to degradation, likely due to increased hydrolysis induced by acidic conditions as a result of the metabolic activity of cells, as well as to a higher porosity and better accessibility of cleavage sites [141,150]. It can be observed that the degradation is affected by porosity, showing a direct relation between the porosity of constructs and degradation percentage. It is widely known that the porosity of scaffolds could influence the degradation rate [151,152]. Zhang et al. demonstrated that increasing the porosity of scaffolds leads to their higher and faster development compared to less porous scaffolds [153]. The results obtained in our work are in accordance with other studies related to cartilage regeneration that reported a low percentage of degradation similar to our data (~between 1 and 7%). Correia et al. reported a slow degradation rate and confirmed that the degradation increased with higher HA concentration [141]. Thus, CBCs based on hMSC-AL/HA-bioink could be used for CTE because in vivo tissue regeneration is a slow process [143].

The electrical properties could help to understand the cellular processes that occur on the surface of the constructs. It has been reported that the ζ can affect both static cell behavior and dynamic cell behavior [151,154]. The negative ζ observed could be attributed to the dominance of hydroxyl and carboxyl groups on the surface of HA and AL [155,156], as well as the presence of terminal carboxylic groups on the surface of PLA [157]. The ζ values obtained for the developed CBCs are in agreement with previous studies, where values gradually decreased with increasing pH, ranging from −2 mV to −35 mV, and recorded −18 mV at pH 7.4 [158]. AC has an intracellular pH of around 7.15 and a slightly acidic extracellular medium, around 6.6 [159]. Under physiological conditions, a value of ζ around 20 is observed. These ζ values mimic the negative charges of the GAG chains on the ECM. Negative charges attract water molecules into the ECM. This fact leads to an increase in the tension of the collagen network and contributes to the tension that the tissue can resist. Further, it will be useful to interact with positively charged proteins [160,161]. Conductivity can influence cell growth and differentiation; therefore, the use of biocompatible conductive polymers could create a favorable culture environment to promote cellular activities [162]. The high values of conductivity obtained in the CBCs based on hMSC-AL/HA-bioink may contribute to induce the specific differentiation of hMSCs, as well as to enhance the cell-to-cell communication and cell adhesion [163].

The elastic modulus of a scaffold could be related to the optimization of the bioprinting process and the homogeneity of the material, as mentioned earlier by Martínez-Moreno et al. [164]. The result obtained from the mechanical test of CBCs shows a relevant Young’s modulus value compared to other biomaterials used in CTE. For example, in the study conducted by Petitjean et al. on healthy and unstressed AC, the elastic modulus of the native tissue varied between 0.25 and 3 MPa [165]. Although CBCs have a slightly higher Young’s modulus (4.069 ± 0.567 MPa), this value suggests that the constructs are suitable for load-bearing in complex clinical applications, offering structural stiffness, functionality, and scaffold integration comparable to that of native tissue.

In summary, results from biological analyses demonstrated the beneficial effect of HA, as CBCs based on hMSC-AL/HA-bioink supported high viability, stimulated cell proliferation, and facilitated the chondrogenesis of hMSCs better than the control construct bioprinted from hMSC-AL-bioink, which is considered as the gold standard bioink for CTE [166,167,168]. From a physicochemical point of view, our CBCs have microarchitecture (morphology and porosity) with sufficient space for cell proliferation and surface characteristics (ζ and conductivity) that are suitable for controlling cell-to-cell and cell-to-material interactions. On the other hand, our results indicate that the developed CBCs exhibit slow degradation, which can be attributed to PLA. This slow degradation is desirable in cartilage tissue regeneration applications. However, modeling the degradation processes in vivo may be needed to understand how they affect tissue regeneration. Furthermore, the low swelling observed in this material during our assay may affect the exchange of nutrients and oxygen through the material structure. Although our results did not compromise cellular viability, the degradation rate and swelling ratio could be improved by modifying the structural conformation, molecular weight, orientation, and crystallinity of PLA [169]. The degradation rate could also be increased by decreasing the molecular weight of the AL. Finally, in the future, we expect to investigate cartilage regeneration in vivo using these hybrid constructs in animal models to evaluate their potential for clinical application.

## 5. Conclusions

In conclusion, our results demonstrated that the use of bioprinted CBCs based on hMSC-AL/HA-bioink and thermoplastic PLA for AC repair could enhance the regeneration and/or formation of hyaline cartilaginous tissue. This is because CBC based on hMSC-AL/HA-bioink has been shown to provide a suitable environment for cell growth, despite it exhibiting similar benefits in terms of functionality to the control construct based on hMSC-AL-bioink, whose chondro-inductive properties, independently of other stimulating factors, have been already evidenced. In addition, the physicochemical properties of the CBCs based on hMSC-AL/HA-bioink have only been slightly altered compared to cell-free CBCs based on AL/HA-bioink, confirming that the artificial cartilage developed in this work could be suitable for use in CTE. To verify these assumptions, future long-term studies in vitro and in vivo should be carried out.

## Data Availability

The original contributions presented in the study are included in the article, further inquiries can be directed to the corresponding authors.
